# Psychosocial Correlates of Diabetes Self-management Practices

**Published:** 2017-06

**Authors:** Muhammad W. DARAWAD, Sawsan HAMMAD, Sultan MOSLEH, Osama A. SAMARKANDI, Ayman HAMDAN-MANSOUR, Amani A. KHALIL, Diana ARABIAT

**Affiliations:** 1. Dept. of Clinical Nursing, School of Nursing, University of Jordan, Amman, Jordan; 2. Dept. of Community Health Nursing, Faculty of Nursing, University of Jordan, Amman, Jordan; 3. Dept. of Adult Health Nursing, Faculty of Nursing, University of Mutah, Karak, Jordan; 4. Basic Science Department, Prince Sultan bin Abdulaziz College for Emergency Medical Services, King Saud University, Riyadh, Saudi Arabia; 5. Dept. of Maternal & Child Health Nursing, School of Nursing, University of Jordan, Amman, Jordan; 6. Dept. of Innovative Research, School of Nursing and Midwifery, Edith Cowan University, Perth, WA, Australia

**Keywords:** Diabetes, Self-management, Depressive symptoms, Social support, Jordan

## Abstract

**Background:**

Self-Management is a crucial regimen for patients with diabetes mellitus. Many factors have affected patients’ self-management practice including psychosocial factors. Literature revealed contradictory results concerning the psychosocial correlates of patients’ self-management practices. Therefore, this study assessed the psychosocial correlates of diabetes self-management practices among Jordanian diabetic patients.

**Methods:**

A descriptive, cross-sectional, correlational design was utilized to collect data (conducted in the middle region of Jordan in 2015) from 341 Jordanian outpatients with diabetes using self-reported questionnaires (Social Support Scale, CES-D, and Summary of Diabetes Self-Care Activities) and chart review.

**Results:**

Participants reported practice rate of 2.85/ 7 (*SD*=1.3), with diet practice the most (*M*=3.66, *SD*=1.5) and exercise the least (*M*=1.53, *SD*=2.1). Participants reported receiving social support (*M*=3.23, *SD*=1.3) less than needed (*M*=3.39, *SD*=1.3). High levels of depressive symptoms were reported (*M*=17.1, *SD*= 11.4). Diet practices had significant positive correlation with family support attitude (*r*= .266, *P*= .000) and negative correlation with depressive symptoms (*r*= − .114, *P*= .037). Testing blood sugar significantly correlated with both support needed (*r*= .144, *P*= .008) and support received (*r*= .166, *P*= .002).

**Conclusion:**

Jordanian DM patients were found to practice less than optimum DM self-management practices, and to consider diet practices than exercise practices. This study confirmed that the subcategories of DM self-care management should be considered rather than considering the general plan.

## Introduction

According to the International Diabetes Federation (1), diabetes mellitus (DM) is a devastating chronic illness that has a prevalence rate of 8.3% worldwide, with a total of 371 million adult patients, 187 million undiagnosed cases, 4.8 million deaths, and $471.6 billion for healthcare expenses. The developing countries suffer higher prevalence rate of diabetes, in which the number of diabetic patients is expected to increase by 69% compared to 20% in the developed countries (2). Jordan, as a developing country, has a high prevalence rate that reached 11.6%, which is higher than the global DM prevalence rate, with annual 2740 deaths (1). This high DM prevalence rate in Jordan can be explained by the demographic and socio-cultural changes such as aging of the population and the emergence of the junk food culture (3), increased the environmental risk factors for diabetes such as the high rates of obesity and physical inactivity (4), and elevated lipid profile (5).

Many consequences have been suffered by the Jordanian diabetics including psychosocial consequences such as poor quality of life and increased depressive symptoms (6), sexual dysfunction (7), and long-term physical complications (3). The seriousness of DM complications, the lack of cure, and the high cost of health care mandate different approaches in dealing with this devastating illness, in which patients’ self-management is considered the key to promoting patients’ health and healthcare services. Further, self-management was considered as the foundation of the chronic care model for patients who are suffering from chronic illnesses (8). Self-management includes activities and lifestyle changes undertaken by individuals for health promotion and management of life with an illness (9).

In DM, self-management is challenging because it necessitates a difficult combination of various aspects of diabetes treatment including adherence to diet and exercise regimen, prescribed medications, foot care guidelines, and frequent blood sugar monitoring. On the other hand, adherence to diet and exercise was found to be associated with low glycated hemoglobin (HbA1c) (10). Similarly, medication adherence was reported to be a significant predictor of glycemic control (11).

Despite the importance of diabetes self-management, literatures have contradictory results regarding diabetic patients’ adherence to self-management guidelines. For instance, diabetic patients were highly adherent to various self-management practices such as blood glucose monitoring, foot care, and exercise practice, diet plans, and medication (12, 13). On the contrary, other studies found diabetic patients to be less adherent to self-management practices (14–16). Further, patients were found to be more adherent to medication and physician appointment than diet and exercise guidelines (17).

The engagement of diabetic patients in self-management activities was affected by many psychosocial factors, among which social support was found to have a direct influence on HbA1c and self-care management (13, 18). Further, social support from healthcare providers and family had strong relationship with the patients’ body mass index (BMI) (19) and was a significant predictor for adherence to diabetic diet and foot care (20, 21). On the other hand, the lack of social support was associated with poor self-care management (22, 23) and nonadherence to medication (24).

Despite the importance of social support for diabetic patients, they reported receiving inadequate support from their social system (25). Further, male diabetic patients reported receiving much support than female patients and reported the spouse to be the primary supporter (26, 27), whereas, the health care provider was the primary supporter for female patients (28).

Depression is another psychosocial factor that was found to be prevalent among diabetic patients. Indeed, they are more likely to have depression than the general population (29). For instance, 24.9% of the diabetic patients had either depression or diabetes-related distress (30). Further, depression affected diabetic patients’ commitment to self-management practices (31) and their glycemic control (32).

The goal of diabetic patients’ management is to achieve a target glycemic control. Patients’ HbA1c was treated as the gold standard measure to monitor patients’ control of their blood glucose level. Despite its objectivity, it does not indicate which aspect of self-care management has the deficiency. Further, review of literature revealed a contradiction in the results of psychosocial correlates of patients’ self-care management activities. This creates unclear description of the effect of these factors on diabetic patients’ self-management and glycated hemoglobin. In addition, Jordanian diabetic patients’ levels of social support and depression, and their relationships to diabetes self-management practices are unknown.

Therefore, the aim of this study was to assess the psychosocial correlates of diabetes self-management practices among Jordanian diabetic patients. The results of this study can be used to set up a baseline that can be used in future interventional studies. Specifically, this study is trying to answer the following research questions:

1. Which of the diabetes self-management practices Jordanian diabetic patients adhere the most?
2. What is the level of social support and depression among Jordanian diabetic patients?
3. What are the relationships among depression, social support, selected demographics, and diabetes self-management practices?
4. What are the differences in Jordanian diabetic patients’ diabetes self-management practices based on their categorical demographics?

## Methods

### Design

A descriptive, cross-sectional, correlational design was utilized, with the use of self-reported questionnaire and chart review to collect some clinical data including participants’ HbA1c and BMI.

This study collected data from Jordanian diabetic patients at diabetes clinics (outpatient departments) of different hospitals representing different healthcare sectors in Jordan (public, private, and educational). Further, a stratification method of sampling technique was used to divide Jordan into three parts (south, middle, and north). A total number of nine hospitals, three from each stratum, were randomly selected.

### Population

The study was conducted in the middle region of Jordan in 2015. The target population of this study includes all Jordanian diabetic patients. A convenience sample of adult diabetic patients was randomly recruited and invited to participate in this study. To be eligible, participants had to: 1) be at least 18 yr old, 2) be diagnosed with DM for at least one year, 3) be free from severe mental, physical, or cognitive deterioration, and 4) accept participation.

To calculate the sample size, the power analysis technique was utilized applying Cohen’s table (33) with medium effect size, Power= .80 and *α*= .05, using Analysis of Variance (ANOVA) test. A minimum sample of 180 diabetic patients was needed to represent Jordanian diabetic patients. However, with an estimation of a 50% response rate, more than 400 participants have been approached and invited to participate in this study.

### Data Collection

After obtaining the required ethical approval, a pilot study was carried out to assess the feasibility of the study and to evaluate the psychometric properties of the instruments. Then, research assistants screened patients for eligibility, and those who were eligible were invited to participate voluntarily in the study. Those who agree to participate were handed a cover letter that illustrated the study title, purpose, significance, and a statement informing the participants with their rights of privacy and confidentiality. The whole study package was in the Arabic language. Data collection was performed in during the patients’ visits to the outpatient units. The average time it took for the interview and to fill out the study instruments was approximately 30–40 min.

### Instruments

The questionnaire package of this study consisted of four sections. The first section asked participants to answer questions regarding their demographic characteristics including their age, gender, marital status, level of education, and, income, household members, duration of having diabetes, blood sugar self-monitoring, and smoking status. Other clinical demographics were retrieved from patients’ charts including co-morbidities, weight and height, Glycated hemoglobin, and type of treatment.

The second section used the Social Support Scale, which is part of the Diabetic Care Profile developed by Michigan Diabetes Research and Training Center (34). This scale consists of three subscales (support need, support received, and family support attitude). Each subscale consists of six items that measure social support from family members or friends regarding different aspects of diabetic patients’ treatment regimen. The three subscales utilize a 5-point Likert scale (1–5); however, the mean scores were utilized in analysis. In addition, this scale asks a multiple-choice question about the primary supporter. To assess participants’ depressive symptoms, the third section used the Center for Epidemiologic Studies Depression Scale (CES-D), which is a widely used, valid and reliable scale (35). The CES-D scale asks participants to report the frequency of occurrence for 20 items during the last week, using a 4-point Likert scale ranging from 0 “*Rarely*” to 3 “*Most or all of the time*”, with a possible range between 0–60 and a score of 16 or more is considered depressed.

The fourth section used the Summary of Diabetes Self Care Activities (SDSCA), which is a valid, reliable, and multidimensional (36). The SDSCA is a self-report measure that assesses four aspects of DM self-management including dietary control (four items), exercise (two items), blood sugar testing (two items), and foot care (two items). The four subscales asked about the average number of days in the last seven days the participant followed the prescribed item. According to the authors’ recommendations, the means of the subscales were entered in the analysis section. The three utilized instruments were translated from English to Arabic using the standard protocol of forward and backward translation. All the researchers are bilingual, therefore, two of them made the forward translation into Arabic. A third researcher made the back-translation from Arabic into English. Two faculty members (colleagues) evaluated the two versions for equivalency of the meaning. Minimal differences were reported and the final wording of the version was agreed upon. In addition, an expert in instrument development was consulted before finalizing the Arabic versions. Further, the participants conducted a pilot study to ascertain that the Arabic version was clear and understandable.

### Ethical approval

The ethical approval to conduct this study was obtained from the Scientific Research Committees at Faculty of Nursing the University of Jordan and the participating hospitals. Participants were asked to read a cover letter stating the study title, purpose, and participants’ rights. Participants’ were assured that their data will be secured, and their identities will be protected through coding their demographic data. In addition, participants were advised that participation is voluntary and would not affect their medical treatment and that they could withdraw from the study with no penalties.

### Statistical analysis

The Social Package for social sciences (SPSS, ver. 17 (Chicago, IL, USA) was to enter and analyze study data. Data screening and cleaning were conducted prior to data analysis process. Descriptive statistics including central tendency measures (means, and medians) and dispersion measures (standard deviation and ranges) were used to describe participants’ demographics and the main study variables. Inferential statistics were used to answer the study questions including Pearson correlation to assess the correlation of diabetes self-management practices with both psychosocial and continuous demographic variable. Besides, to assess differences in diabetes self-management practices based on categorical demographic variables, a series of students’ *t-*tests for independent groups and analysis of variance (ANOVA) tests were conducted.

## Results

Out of 411 distributed questionnaires, 341 were returned (a response rate of 83%). The majority of the sample was females (52.5%), married (75.4%), nonsmokers (78%), educated with less than high school (75.4%), frequently monitor their blood sugar (88%), and mostly treated with oral hypoglycemic agents (48.4%). The average of the participants’ age was 55.2 yr (*SD*=12.9), household members 4 (*SD*=2.4), income 650 USD (*SD*=425), duration of illness 11.3 yr (*SD*=9.2), BMI 29.7 (*SD*=5.5), and A1C level 7.9 (*SD*=1.9). Detailed description of the sample characteristics is shown in Table 1.

**Table 1:** Sample Demographic Characteristics (N= 341)

**Characteristics**	**Mean (SD)**	**n (%)**
**Gender**		
Male		162 (47.5)
Female		179 (52.5)
**Marital Status**		
Single		20 (5.9)
Married		257 (75.3)
Others		64 (18.8)
**Current Smoker**		
Yes		75 (22)
No		266 (78)
**Education**		
Less than secondary school		243 (71.3)
Diploma		45 (13.2)
Bachelor or higher		51 (15.5)
**Diabetes Treatment**		
Insulin		96 (28.2)
Oral tablet		165 (48.4)
Insulin + Oral tablet		78 (23.4)
**Blood Sugar Self-Monitoring**		
Yes		300 (88)
No		41 (12)
**Age** (yr)	55.2 (12.9)	
**Body Mass Index**	29.7 (5.6)	
**Household members**	4 (2.4)	
**Income (**USD**)**	630 (425)	
**Duration of illness** (years)	11.3 (9.2)	
**Glycated Hemoglobin**	7.9 (1.9)	
**No. of Cigarettes/ day**	4.4 (10.1)	

To answer the first question regarding which of the diabetes self-management practices Jordanian diabetic patients adhere the most, participants reported being mostly adherent to diet practices (*M*=3.66, *SD*=1.5), followed by foot care (*M*=3.25, *SD*=2.7), whereas exercise was the least (*M*=1.53, *SD*=2.1).

The total adherence rate was 2.85 (*SD*=1.3). The individual item that had the highest adherence rate was “*eating low-fat food*” (*M*=4.19, *SD*=2.0), whereas the item that had the lowest adherence rate was “*participation in a specific exercise session*” (*M*=1.16, *SD*=2.1). Table 2 shows detailed item analysis of the diabetes self-management practices. Regarding the level of social support and depression among Jordanian diabetic patients (the second research question), participants reported receiving social support (*M*=3.23, *SD*=1.3) less than needed (*M*=3.39, *SD*=1.3). Meanwhile, the mean family attitude toward social support was 3.97 (*SD*= .68). The spouse was the main social support provider for the general sample (49%), followed by another family member (39%), whereas healthcare professionals were the least (5.6%).

**Table 2:** Item Analysis of Diabetes Self-Management Practices

	**Items How many of the last SEVEN DAYS have you followed:**	Mean^*^ (SD)	**Percent of commitment (> 3 days/week)**
	**Diet**	3.66 (1.5)	
	A healthful eating plan	3.58 (2.6)	52.5
	Your eating plan	3.79 (2.5)	56.9
	Eating fruits and vegetables	3.09 (2.4)	43.8
	Eating low fat foods	4.19 (2.0)	69.8
	**Exercise**	1.52 (2.1)	
	Participation in at least 30 minutes of exercise	1.89 (2.4)	23.5
	Participation in a specific exercise session	1.16 (2.1)	14.4
	**Blood Sugar Test**	2.16 (2.4)	
	Testing your blood sugar	2.34 (2.6)	27.3
	Testing your blood sugar as recommended	1.96 (2.5)	22.3
	**Foot Care**	3.25 (2.7)	
	Checking your feet	3.55 (3.1)	49.4
	Inspecting the inside of your shoes	2.94 (3.1)	40.3
	**Total**	2.85 (1.3)	

* Range= 0–7

For male patients, the spouse was the main social support provider (71.6), whereas for female patients another family member (59.8%). Further, the level depressive symptoms was 17.1 (*SD*= 11.4). Detailed description of the main study variables is provided in Table 3.

**Table 3:** Descriptive Statistics of the Main Study Variables

**Characteristics**	**α**	**Mean (SD)**	**Minimum**	**Maximum**
Social Support: Needed	.88	3.39 (1.3)	1	5
Social Support: Received	.88	3.23 (1.3)	1	5
Social Support: Family Attitudes	.63	3.97 (0.7)	1.83	5
Depression	.89	17.1 (11.4)	0	54
Self Management: Diet	.61	3.66 (1.5)	0	7
Self Management: Exercise	.79	1.52 (2.1)	0	7
Self Management: BS Test	.89	2.17 (2.4)	0	7
Self Management: Foot Care	.70	3.25 (2.7)	0	7
Self Management: Total	.66	2.85 (1.3)	0	6.30

BS: Blood Sugar

To examine the relationships of diabetes self-management practices with selected psychosocial and demographic variables, Pearson correlation test was used (Table 4). Diet practices was found to have a significant positive correlation with family support attitude (*r*= .266, *P*= .000) and negative correlation with depression (*r*= − .114, *P*= .037). Testing blood sugar significantly correlated with both support needed (*r*= .144, *P*= .008) and support received (*r*= .166, *P*= .002). Finally, total self-management practices positively correlated family support attitude (*r*= .197, *P*= .000), negatively correlated with depression (*r*= − .121, *P*= .028). Exercise and foot care practices did not significantly correlate with any of the psychosocial variables.

**Table 4:** Correlates of Diabetes Self-Management Practices

	**Self-Management Practices**				
**Variable**	**Diet**	**Exercise**	**Blood Sugar Test**	**Foot Care**	**Total**
Support Needed	−.099	.069	.144^**^	−.058	.004
Support Received	−.064	.031	.166^**^	−.066	.016
Family Support Attitudes	.266^**^	.036	.063	.072	.197^**^
Depression	−.114^*^	−.056	−.035	−.071	−.121^*^
Age	.115^*^	−.236^**^	−.054	.062	.022
Income	.110^*^	.062	−.002	.029	.051
Body Mass Index	−.044	−.145^**^	−.041	.010	−.094
HbA1C	−.123^*^	−.078	.151^**^	−.058	−.051
Duration of Illness	.034	−.099	.078	.018	.022
Household members	.070	.218^**^	.017	.061	.065

Note: Pearson Correlations

** Correlation is significant at α=0.01 (2-tailed),

* Correlation is significant at α=0.05 (2-tailed)

Regarding the demographic variables, diet management practices were better among patients who were older (*r*= .115, *P*= .034) and with higher income (*r*= .110, *P*= .042), whereas exercise practice was better among patients who were younger (*r*= −.236, *P*= .000), with less BMI (*r*= − .145, *P*= .007), and with more household members (*r*= .218, *P*= .000). Blood sugar testing, foot care, and total self-management practices were not found to correlate with any of the participants’ demographics. Finally, lower HbA1c readings were noted among patients with higher diet practices (*r*= −.123, *P*= .032) and less frequent blood sugar testing (*r*= .151, *P*= .009).

A series of t-test and ANOVA test were conducted to test differences in diabetes self-management practices based on categorical demographic variables (Table 5).

**Table 5:** Analysis of Diabetes Self-Management Practices based on categorical participants’ demographics

	**Self-Management Practices**				
**Variable**	**Diet**	**Exercise**	**Blood Sugar Test**	**Foot Care**	**Total**
**Gender**					
Male	3.61	1.85^**^	2.32	3.24	2.93
Female	3.70	1.23	2.02	3.26	2.78
**Marital Status**					
Single	3.28	2.45^*^	3.73^**^	2.80	3.11
Married	3.62	1.60	2.08	3.29	2.84
Others	3.94	1.0	2.03	3.23	2.82
**Education**					
Less than high school	3.56	1.32^**^	1.98^*^	3.08	2.70^**^
Diploma	4.15	1.78	2.23	3.56	3.17
Bachelor or higher	3.69	2.29	2.99	3.79	3.28
**Diabetes Treatment**					
Oral tablet	3.82	1.83^**^	1.47^**^	3.26	2.84
Insulin	3.48	1.40	3.15	3.06	2.92
Insulin + Oral tablet	3.56	.91	2.55	3.44	2.80
**Current Smoker**					
No	3.67	1.40	2.27	3.35	2.87
Yes	3.61	2.0^*^	1.77	2.89	2.80

** Correlation is significant at α=0.01 (2-tailed),

* Correlation is significant at α=0.05 (2-tailed)

Exercise was found to be better among participants who were male, single, smoker, educated with bachelor degree or higher, and treated with oral tablet only. Similarly, blood sugar testing was more frequent among participants who were single, educated with bachelor degree or higher, and treated with insulin only. Finally, those educated with less than high school had less total self-management practices than their counterparts.

## Discussion

The current study examined Jordanian DM patients’ self-management practices and their psychological correlates (social support and depression) along with their correlates with selected demographics. A major strength of this study is the use of the SDSCA, proved valid, reliable, multi-dimensional, and the most widely used self-report instrument to assess diabetic patients self-management practices (36). Further, the subscales of the SDSCA were used according to the recommendations by Johnson (37) who recommended that due to the multidimensionality of DM self-care, components of DM self-care should be separately assessed rather than combining across components. Participants’ total practice of DM self-management practices was 2.85 d out of 7 (40.7%). This low practice of self-care is much less than an average of 4.27 among DM patients in Tanzania (15). However, both figures are considered suboptimal indicating the difficulty of following strict self-care practices in chronic illnesses in general (38, 39). The subcategories of DM self-care were considered per recommenda-tions. Examining the subscales of self-management practices revealed that participants practiced diet practices the most (3.66), whereas the least practice was to exercise practices (1.53). A considerable consistency was found across seven studies with DM patients typically reporting higher diet than exercise self-practices (36). Similarly, exercise was the least self-care activity carried out by DM patients in their study (40). Like other chronic illnesses, this nonadherence confirms that Jordanian DM patients are at a great risk for developing long-term complications (41). Therefore, diabetic patients should be encouraged to adhere more to all self-management practices to prevent developing those complications.

As a permanent problem, diabetic patients continue to report receiving less social support (3.23 out of 5) than they actually needed (3.39). The same lack of sufficient social support was reported among diabetic patients worldwide (25). Such lack of support made 45.2% of DM patients to seek support occasionally and frequently (42). The most common social support provider was the spouse for male participants and another family member for female patients. Having the spouse to be the main social support for male patients is similar to the literature (26,27), but having other family member to be the primary supporter for female patients is opposite (28), explained by the Arabic and Islamic culture in which taking care of the elderly is a privilege for the youths to show respect and concern. Health-care professionals were the least to provide social support for their patients. Healthcare professionals may lack the appropriate knowledge to deal with their patients (43), and that they should be engaged in supporting their patients, which can positively encourage the patients toward better self-management practices. Tailored educational programs for healthcare professionals could be of great benefit in this regard (44).

Concurrently, participants reported having elevated levels of depression (M=17+− 11.4), which exceeds the cut-off point considered for depressed patients (35), and confirms the presence of the depressive symptoms among patients with chronic illnesses in general (38) and diabetic patients in specific (6,29). Therefore, Psychological screening for depressive symptoms is encouraged before initiating health education programs or management plans for DM patients.

Examining the psychological correlates of DM self-management practices revealed that those who received support that is more social practiced more frequent blood sugar testing, and those whose families had attitudes that are more positive practiced more frequent diet and total self-management practices. This highlights the importance of including social support in diabetic patients’ self-management plans, found to have a direct influence on their self-management practices (18, 21). The effect for getting sufficient social support was found among patients with other chronic illnesses (38). Conversely, depressive symptoms had a negative correlation with participants’ diet and total self-management practices. The same correlation was reported among diabetic patients worldwide (31, 32). Therefore, any educational program for diabetic patients to enhance their self-management practices should take into consideration the presence of depressive symptoms and the lack of sufficient social support as inhibiting factors.

Regarding the demographic correlates of DM self-management practices, older patients and those with higher income practiced more frequent diet practices. On the other side, younger patients, patients with lower BMI values, and those with larger household members practiced more exercise than their counterparts did. These results echo those reported in literature (32, 40). The characteristics of patients of patients who practiced fewer self-management practices should be taken into consideration while developing patients’ management plans. However, none of the demographic variables correlated with total self-management practices, which confirms that the subcategories of DM self-care management should be considered rather than considering the general plan.

## Conclusion

Jordanian DM patients were found to practice less than optimum DM self-care practices and to consider diet practices than exercise practices. Further, participants were found to receive less social support than perceived needed and to have high levels of depressive symptoms, for which psychological screening is highly encouraged before initiating management plans and health education programs. Some gender differences were correlated with participants’ self-care practices that are highly encouraged to be considered. Finally, the subcategories of DM self-care management should be considered rather than considering the general plan.

## Ethical considerations

Ethical issues (Including plagiarism, informed consent, misconduct, data fabrication and/or falsification, double publication and/or submission, redundancy, etc.) have been completely observed by the authors.
